# Cellular cardiac electrophysiology modeling with Chaste and CellML

**DOI:** 10.3389/fphys.2014.00511

**Published:** 2015-01-06

**Authors:** Jonathan Cooper, Raymond J. Spiteri, Gary R. Mirams

**Affiliations:** ^1^Computational Biology, Department of Computer Science, University of OxfordOxford, UK; ^2^Numerical Simulation Research Lab, Department of Computer Science, University of SaskatchewanSaskatoon, SK, Canada

**Keywords:** CellML, cardiac, ODE, electrophysiology, simulation, C++, software

## Abstract

Chaste is an open-source C++ library for computational biology that has well-developed cardiac electrophysiology tissue simulation support. In this paper, we introduce the features available for performing cardiac electrophysiology action potential simulations using a wide range of models from the Physiome repository. The mathematics of the models are described in CellML, with units for all quantities. The primary idea is that the model is defined in one place (the CellML file), and all model code is auto-generated at compile or run time; it never has to be manually edited. We use ontological annotation to identify model variables describing certain biological quantities (membrane voltage, capacitance, etc.) to allow us to import any relevant CellML models into the Chaste framework in consistent units and to interact with them via consistent interfaces. This approach provides a great deal of flexibility for analysing different models of the same system. Chaste provides a wide choice of numerical methods for solving the ordinary differential equations that describe the models. Fixed-timestep explicit and implicit solvers are provided, as discussed in previous work. Here we introduce the Rush–Larsen and Generalized Rush–Larsen integration techniques, made available via symbolic manipulation of the model equations, which are automatically rearranged into the forms required by these approaches. We have also integrated the CVODE solvers, a ‘gold standard’ for stiff systems, and we have developed support for symbolic computation of the Jacobian matrix, yielding further increases in the performance and accuracy of CVODE. We discuss some of the technical details of this work and compare the performance of the available numerical methods. Finally, we discuss how this is generalized in our functional curation framework, which uses a domain-specific language for defining complex experiments as a basis for comparison of model behavior.

## 1. Introduction

Thousands of studies have been performed with computational cardiac action potential models to investigate the electrophysiological mechanisms governing the activity of the heart. There are now over 100 different models, each representing different hypotheses about how the action potential is generated from underlying ionic currents and how it adapts to different situations. It is advantageous to be able to compare and contrast these models (i.e., hypotheses) in an automated and consistent manner.

A solution to this challenge requires a representation of the models in a form that facilitates such comparison. For each model, we want one reference encoding of it that is used to generate both the equations and parameter details in the published paper as well as the programming code used to simulate the model, in order to ensure these describe the same mathematics. The CellML standard (Lloyd et al., 2008) provides us with such a format. It is an XML-based language, and hence computer processable, with tools available for editing, simulating, and presenting models (Garny et al., 2008). There is also a public repository of models encoded in CellML (Yu et al., 2011) that includes the majority of published cardiac cellular electrophysiology models. This repository is thus a source of reference model versions that have been through a curation process.

This article covers the features available in Chaste v3.3 for exploiting CellML to do cardiac electrophysiology research. Some of the capabilities were available in earlier releases and have been described elsewhere, particularly in Garny et al. (2008), Cooper and McKeever (2008), Cooper (2009), Pathmanathan et al. (2010), Cooper et al. (2011a), Marsh et al. (2012), and Mirams et al. (2013). While describing Chaste's current capabilities, we therefore focus on new (and previously undescribed) features.

Almost all cellular electrophysiology models follow the same basic assumption, following the ground-breaking work of Hodgkin and Huxley (1952). The cell membrane is modeled as an ideal capacitor; i.e., the transmembrane voltage *V* obeys the following ordinary differential equation (ODE):

(1) $$\frac{\text{d}V}{\text{d}t}=-\frac{1}{C_{m}}(\sum_{j}I_{j}+I_{\text{stim}}),$$

where *t* is time, *C_m_* is the capacitance of the membrane, *I_j_* represents ionic current *j*, and *I*_stim_ is an applied stimulus current.

There are typically many other variables in the system (denoted “**u**”) that govern the currents *I_j_*. These variables describe ion-channel states and other quantities such as ion concentrations in different cellular compartments. These satisfy the ODE system

(2) $$\frac{\text{d}u}{\text{d}t}=f(u,\;V),$$

where **f(u**, *V*) is a vector of functions describing the evolution of each component of **u**. Equations (1, 2) can therefore represent any typical ODE system defined by a CellML electrophysiology model.

Mostly, this paper considers single cell electrophysiology simulations, but all of the models and solvers are also available to use in the “ODE solving” portion of cardiac tissue simulations. Indeed, some solvers are developed specifically for this context, and so results for this setting are included. We present only the simplified monodomain equations for modeling tissue level behavior; see Keener and Sneyd (1998) for more details. These take the form of a coupled system of partial differential equations (PDEs) given by:

(3) $$\chi \left(C_{m}\frac{\partial V}{\partial t}+I_{\text{ion}}(u,\;V)\right)-\nabla \;\cdot \;\left(\sigma \nabla V\right)=0,$$

(4) $$\frac{\partial u}{\partial t}=f(u,\;V),$$

where: *V*, **u**, *C_m_*, and **f** are as defined above; *I*_ion_ = ∑*_j_I_j_* is the total ionic current; χ is the membrane surface area per unit volume; and σ is a conductivity tensor.

In the next section, we detail Chaste's capabilities. Then in Section 3, we present their effectiveness in various scenarios. In Section 4, we discuss key features arising and outline some future directions.

## 2. Methods

CellML provides us with a structured XML encoding of the mathematical equations defining a cellular model, along with metadata. This is not a form that can be directly solved. Instead, all tools that perform simulations of models encoded in CellML (see (Garny et al., 2008), for a list) convert the equations into computer code in some programming language and apply a numerical method for their solution. Because Chaste is written in C++, we convert CellML to C++ code using a toolkit called PyCml (Cooper, 2009), distributed as part of Chaste. PyCml therefore generates C++ code designed to interact with Chaste, and this process is described in the first subsection. Chaste provides a variety of solvers for the ODE systems that comprise cellular models, detailed in Section 2.2. Finally, we describe some additional features of Chaste that are useful for working with these models in Section 2.3.

### 2.1. Conversion of CellML

Detailed user documentation for the CellML conversion process is provided as part of the Chaste wiki at https://chaste.cs.ox.ac.uk/q/cellml-guide. Here, we shall focus on a high-level description of the techniques used and features available. Using CellML provides us with a source for curated encodings of cellular electrophysiology models in a computer-processable format. However, as we have indicated in earlier work (Cooper et al., 2011a), this does not necessarily make such encodings easy to use. Our software makes use of various techniques to provide straightforward simulation and analysis capabilities.

#### 2.1.1. Metadata annotation

Central to Chaste's CellML handling is the use of *ontological annotation* (Beard et al., 2009; Wimalaratne et al., 2009) to ensure consistent identification of biological entities within different models. An ontology describes a collection of concepts or entities, and the relationships between them, in a way that allows computers to make logical inferences about the things described. Ontological annotations are implemented using the Resource Description Framework (RDF), which represents “knowledge” as triples of terms: subject, predicate, and object (e.g., “red” “is a” “color”). Each variable within a model, or the whole model itself, may be used as the subject in such a triple, with terms from one or more ontologies providing the predicate and object. At present, we mainly use this feature to provide standard naming for concepts such as the transmembrane potential, ionic currents, and their maximum conductances, with the object terms taken from our own ontology developed for this purpose. (See https://chaste.cs.ox.ac.uk/q/cardiac-metadata for the ontology contents.) The predicate used in these cases represents the “is” relationship; annotations thus state “*this variable is the transmembrane potential*,” for instance. The various uses of the annotations are discussed as they occur below.

#### 2.1.2. Standardized interfaces

Within Chaste, time is always measured in milliseconds, voltage in millivolts, and so on, regardless of the convention used by the original cellular electrophysiology model. The code generation process therefore creates C++ code that includes conversions to expose the concepts it requires (time, transmembrane potential, stimulus current, and total membrane ionic current) in consistent units (Cooper et al., 2011a). If given a CellML file without any metadata annotations, PyCml tries to infer which variables represent these core concepts by trying a small selection of common names for *V, C_m_*, and *I*_stim_, specified in its global configuration file, and then analyses the model to identify Equation (1) and hence determine other ionic currents.

However, there are additional benefits to spending the small amount of time required to annotate models with standard names, beyond the minimum required by the conversion process, because these annotations enable other functionality. Chaste provides interfaces to retrieve (or set) model variables by name. If variables have been annotated, the standard names can be used to retrieve them, meaning the same name can be used for the same biological concept no matter which model is being used. This makes it exceptionally easy to run the same simulated experiment on different models. For example, scaling the conductance of a particular ion channel becomes a one-line code addition, regardless of the model choice.

Via the method SetAnyVariable, the value of a variable can be changed regardless of whether it appears as a state variable of the system or is a constant parameter. Similarly, the method GetAnyVariable allows a quantity to be read, whether it be a state variable, parameter, or a “derived quantity” computed as a function of the state variables and parameters. This provides an additional mechanism supporting the analysis of a range of models, because the same biological concept may be treated differently in different models. A common example is an ionic concentration, such as the cytosolic calcium concentration. In most modern models (DiFrancesco and Noble, 1985 onwards), this is a state variable defined by its own ODE. In earlier or simplified models, it may be a fixed parameter (Bernus et al., 2002), or it may be a derived quantity (Matsuoka et al., 2003; Livshitz and Rudy, 2007). By calling GetAnyVariable(“cytosolic_calcium_concentration”), the value can be returned whatever formulation the particular model uses for the calcium system.

PyCml also supports its own metadata annotations to specify which particular variables are of interest, with predicates “modifiable-parameter” and “derived-quantity.” There is also a convenience option to the code generation system called “−−expose-annotated-variables,” which implicitly considers any variable annotated with a standard name to be of interest, marking them as parameters or derived quantities as appropriate (note that state variables are always made available and do not need additional annotation).

A few other PyCml-specific annotations are recognized, notably the ability to set expected ranges for variables that are checked during simulation (for instance, declaring that a concentration should not become negative or that a gating variable that represents a probability should lie between zero and one). Users may also indicate that a model is self-excitatory, and so Chaste should not expect to find a stimulus current.

#### 2.1.3. Stimulus current

Chaste allows a range of stimulus current functions to be applied to models, and typically when creating an instance of a model, a function must be supplied. The decision to do this arises in part from the tissue simulation use case, where stimulation is handled at a higher level, but it is sensible to abstract the externally applied stimulus from the model of the cellular electrophysiology, as we discuss later. After the units conversions, one common stimulus function often suffices to trigger an action potential in any model (Cooper et al., 2011a). However, it can be of benefit to apply the stimulus function defined within the CellML encoding, if there is one. If this stimulus is a “square wave” and its parameters [amplitude, duration, period, start time, and (optionally) end time] have been annotated with our standard names for these concepts, then a method is provided to use a Chaste RegularStimulus with the same parameters. These “default CellML stimuli” are used for our timing tests below to simulate for a fixed number of paces as defined by the stimulus period given in the original model, rather than for a fixed number of seconds.

#### 2.1.4. Dynamic loading

Another feature of Chaste's CellML support that is fundamental to providing easy use of different models is the way in which the code generation process is integrated into Chaste's build system. At one level, this means that a CellML file may be considered as “source code” in the same way as a C++ source file, and if present in a Chaste source folder, it is automatically converted into C++ code and made available for use by other code. By default, this automatic conversion provides both standard and optimized versions of the model, suitable for solving with most of the solvers mentioned in Section 2.2, although this can be customized using per-model configuration files. More powerfully, however, this build process may be employed *while code using Chaste is running*, compiling CellML models and linking them into a running executable on the fly. This allows the choice of model and any code generation parameters to be changed at run time, rather than fixed when the software is built. In the results section, we use this capability to run performance tests on all CellML models available in a folder, iterating over available models and solvers within loops and applying exactly the same analysis to each model/solver combination.

Finally, Chaste provides support for checkpointing long-running simulations, so that they may be resumed from a previously saved (“checkpointed”) state. This is particularly useful for tissue scale simulations, but it can also be relevant for large parameter sweeps or more complex single cell protocols. Models generated dynamically from CellML are fully integrated into this process: their state can be saved just like all other objects within a Chaste simulation, and they can be reloaded. The only additional requirement is that the generated code for the cellular model is still available in the same location when the checkpoint is loaded, because the same code needs to be linked in at run time in order to recreate the same cell model object.

### 2.2. Numerical methods

Many methods are available for solving systems of ODEs (see e.g.,(Iserles, 1996). The ODEs comprising cardiac cellular models are typically stiff, with significant activity in certain variables over short timescales (mainly during the upstroke) and many variables changing over much longer timescales. Solvers available within Chaste, both general and specialized for such systems, are discussed first. We then consider additional techniques that we have implemented for optimizing computational performance. Because some aspects of this, focussing on a tissue context, were discussed in Pathmanathan et al. (2010), here we focus on the novel features introduced and provide a more thorough comparison of performance. We conclude this section with a brief discussion of how things change in the tissue context.

#### 2.2.1. Available solvers

The core Chaste libraries contain a range of basic ODE solvers for initial-value problems that may be applied to cardiac models. These include the forward Euler method (“FE”), as well as second- and fourth-order Runge–Kutta methods (“RK2” and “RK4”). However, due to the stiff nature of the ODEs, such explicit methods are not well suited for use in single cell simulation, requiring prohibitively small timesteps to be numerically stable (and accurate), as shown in Section 3. Implicit schemes are more appropriate for such systems. Furthermore, adaptive timestepping is often necessary for computational efficiency in practice.

Rather than implement implicit ODE solvers or adaptive timestepping directly, Chaste utilizes the CVODE library (Cohen and Hindmarsh, 1996). A wrapper around this library simplifies its usage, presenting a similar interface as for the other ODE solvers in Chaste. The notable differences are that the “timestep” parameter is treated as a maximum CVODE timestep and that there are additional methods available for setting error tolerances. CVODE uses its own data type for representing the vector of state variables in an ODE system, whereas Chaste normally uses the C++ standard template library's vector type. Because converting between these data structures would require copying data at every evaluation of the right-hand side of the ODE system, PyCml generates code specialized for solving with CVODE that uses CVODE's data types internally. Conversions between vector types are then only required when interfacing with external code, and this happens much less frequently.

Chaste also includes numerical methods that are specialized for solving cellular models in the context of a tissue simulation. These exploit the fact that the transmembrane potential *V* is updated as part of the PDE, following Equation (3) instead of (1), and is treated as fixed when solving the ODEs of Equation (4). We include a backward Euler (“BE”) scheme (Whiteley, 2006, 2007; Bernabeu et al., 2009), the Rush–Larsen method (“RL” (Rush and Larsen, 1978), and some generalized Rush–Larsen (“GRL”) methods (Marsh et al., 2012). A key point common to these schemes is that they require changes to the code generation process, transforming the model equations symbolically to fit the requirements of these solvers. These transformations are all performed entirely automatically by PyCml, triggered by supplying appropriate flags specifying the type of solver to be used.

The Rush–Larsen method is a numerical method for ODEs that partitions the variables according to whether they are gating variables or not. The ODEs for the gating variables are linear when *V* is held constant and hence can be solved exactly. The Rush–Larsen method applies this exact formula to advance the gating variables and the forward Euler method to the remaining ones. It is generally quite an effective numerical method for solving cell model ODEs, and this corresponds to the stiffness in the ODEs being captured by the gating variables (Marsh et al., 2012).

However, the stiffness in a cell model is not always captured by the gating variables. In such cases, a more effective solver may be based on the generalized Rush–Larsen method. This method linearises all the ODEs about the current state and integrates the ensuing linear equations exactly. Although significantly more expensive per step than the Rush–Larsen method, the added expense can be offset by the increase in stable step size. More examples and details can be found in (Marsh et al., 2012).

#### 2.2.2. Analytic jacobians

As discussed in Whiteley (2006), using the specialized backward Euler solver requires calculating a Jacobian matrix for the Newton iteration (for a subset of the variables corresponding to a non-linear backward Euler update step). As of Chaste release 2.1, PyCml gained the ability to use Maple (Monagan et al., 2005) to compute an analytic form for this Jacobian, which is then incorporated in the generated C++ code (Bernabeu et al., 2009).

An analytic Jacobian of the full ODE system can be used by CVODE in place of multiple calls to the RHS that are needed to generate a numerical approximation. This often gives an improvement to both accuracy and efficiency. The Jacobian matrix of the ODE system has entries defined as

(5) $$J_{ij}=\frac{\partial f_{i}(v)}{\partial v_{j}},$$

where **v** = {*V*, **u**}, and *i* and *j* range over the number of ODEs in the system [to include *V* and form a full Jacobian for (1) and (2)]. In release 3.2 of Chaste, we added the capability to compute this Jacobian, also using Maple. (Future work will look at using an open-source alternative package, such as SymPy.) Because the entries in the Jacobian matrices contain many common sub-expressions, arising from taking the derivatives of the same complex expression with respect to different variables, we now also take advantage of Maple's ability to do expression simplification and extract such shared calculations into temporary variables, which are therefore evaluated once only. To give an example, the auto-generated analytic Jacobian code for the model of O'Hara et al. (2011) uses over 4000 temporary variables.

However, one caveat is that for some models the analytic Jacobian contains large exponents, which can give non-numerical values when evaluated. As an example, the model introduced in Hund and Rudy (2004) contains the equation

(6) $$ri_{\infty }=\frac{1}{1+\exp\left(\left({\left[Ca^{2+}\right]}_{ss}-0.0004+0.002cafac\right)/0.000025\right)}.$$

The exponent has the potential to become large (because it is divided by 0.000025), and in fact when simply simulating for a single pace, the exponential term exceeds double precision and is represented as “Inf” in memory. The IEEE floating-point arithmetic rules mean that *ri*_∞_ = 1/(1 + Inf) = 0, and so this does not cause a problem when evaluating the right-hand side. However, when derivatives are taken to form the analytic Jacobian, this is no longer the case, and entries of the Jacobian become equal to ‘Inf’.

In fact, most of the models that have unstable analytic Jacobians (e.g., (Livshitz and Rudy, 2007; Benson et al., 2008; Davies et al., 2012) inherit equations such as (6) directly from the Hund and Rudy (2004) model. For such models, we can either avoid computing the analytic Jacobian when generating code or force use of the numerical approximation instead with a method call at run time (as happens by default if no analytic Jacobian is available). The numerical algorithm used by CVODE to approximate the Jacobian uses multiple calls to the right-hand side, and so avoids the issue of non-numerical values in the Jacobian itself. In the rest of the article, we refer to the CVODE using a numerical approximation to the Jacobian and using an analytic Jacobian as the “CVODE NJ” and “CVODE AJ” solvers, respectively.

#### 2.2.3. Partial evaluation

Partial evaluation involves factoring out common parts of a calculation and performing them just once, instead of each time they appear in the equations. For example, an expression like *F/RT* may appear in many thermodynamic calculations and can be computed just once per model. There is no numerical approximation involved here; the partial evaluation simply assists the compiler in optimizing the calculation. Thus, partial evaluation is always utilized in the generated code.

Building on our original implementation (Cooper et al., 2006; Cooper, 2009), in recent releases of Chaste this optimization (and the use of lookup tables described below) can now be applied to all generated code, including the Jacobian matrices, and rearranged mathematics employed by solvers such as backward Euler or Rush–Larsen. They are thus effective for all kinds of model/solver combinations.

#### 2.2.4. Lookup tables

A lookup table is simply a vector of pre-computed values for an expensive function. For example, exp(*V*) may be evaluated for *V* in the range −100 to +80 mV in fine steps, just once, to form a “lookup table.” Then, instead of computing exp(*V*) each time it occurs in the ODE system, we simply interpolate a value from the pre-computed lookup table. A small numerical approximation error is therefore made when using lookup tables, so they are an optional feature.

A new extension to this feature is that tables can be calculated for variables other than *V*: the configuration for what variables may index tables has been linked to the standard name annotations, and table parameters therefore also have their units specified. This means that a single table specification (limits and refinement level) may be applied to any model possessing the relevant annotation, making it easier to provide a widely useful default configuration. The results presented in the next section use the same settings for all models. Although a single table is still only ever indexed by a single variable (because otherwise the interpolation in 2D or higher would become much more complex and the tables much larger, thus reducing the effectiveness of the technique), this allows a greater proportion of the model equations to be replaced by table lookups.

The presence of singularities [e.g., at *V* = 40 when a denominator takes the form (1 − (*V* − 40))], whether in the model equations themselves or in the Jacobians, also causes problems when using lookup tables. Although in normal simulations, the precise singular point of *V* = 40mV is unlikely to be evaluated, when pre-computing lookup tables, this becomes much more likely. This was discussed briefly in Cooper et al. (2006), where analysis of the model equations and Taylor expansions were used to replace some singularities with a suitable value. Unfortunately, in the Jacobians we often get singularities where the equation does not tend to the same value from each direction, instead tending to positive or negative infinity. We therefore employ an ad-hoc approach to work around this, computing lookup table values slightly offset from whole numbers, thus avoiding evaluation exactly at typical singularities. However, when using analytic Jacobians and lookup tables, we have noted that a fine lookup table resolution is often needed to maintain a desired accuracy.

#### 2.2.5. Tissue simulations

Almost all codes that solve the mono/bidomain PDEs use operator splitting numerical schemes (Pathmanathan et al., 2012). Because the PDE formulation assumes that the variables dictating ionic currents vary smoothly in space, the domain is divided into many small volumes, in which it is assumed that those variables are spatially uniform. The PDE is solved over small timesteps to provide *V* in each volume. Each small volume then independently solves Equation (4) [or equivalently, after operator splitting, the set of ODEs (2)] governing the ionic currents, treating *V* as a fixed parameter dictated by the PDE solution. New ionic currents, established by evaluating just the right-hand side of equation (1), then provide an input into the mono/bidomain PDE (Equation 3). The PDE in turn treats these ionic currents as fixed in each volume over the PDE timestep as *V* is updated. To progress through time, the scheme then iterates between updating ODEs and the PDE across each PDE timestep.

This leads to different considerations that may change the most suitable solver for the action potential models:

- the ODE timestep is limited by the PDE timestep (in addition to stability and accuracy);
- Rush–Larsen-style schemes can take advantage of the discretisation approximation of fixed voltage *V* to provide a fast analytic solution to the subset of equations (2) that are (now) linear;
- the ODE solver has to stop and re-start efficiently; and
- any memory overhead associated with solving the ODEs is replicated many times across the whole domain because an independent ODE solver is associated with each small volume.

The latter two points are issues for CVODE, which maintains an internal state to determine the most appropriate step size to use. If state variables of the system change between calls to CVODE, its internal state must be reset. Failure to do so can result in inaccurate solutions or the solver failing to converge. In the single cell context, the voltage *V* is a state variable, and if it were still considered as such when solving the PDEs, a reset would be required at every PDE timestep because *V* is set from the solution to the PDE. However, multiple reset calls to CVODE result in considerable overhead. Instead, we treat *V* as a fixed parameter of the ODE system (not a state variable) when calling CVODE in the tissue context.

Because the *V* parameter changes at each call, a slight approximation is introduced into some of the information that CVODE is storing about the system (e.g., **f** and **J** at the initial conditions were evaluated at the previous value of *V*). Resetting would solve the specified numerical problem slightly more accurately, but by not resetting CVODE, we simply change the update of *V* from occurring at time “*t*” to occurring at “*t* + ϵ,” where ϵ is small. In practice, ϵ is made as small as is required to meet CVODE integration tolerances. As timesteps are refined, we still expect to converge to the same solution that we would achieve when resetting CVODE at each PDE timestep. In the results section, we will see that in practice acceptable accuracy is achieved without resetting CVODE.

### 2.3. Additional features

An advantage of studying cellular electrophysiology within the context of a larger software suite such as Chaste is that additional functionality is available and can be exploited. Notably in this case, Chaste is designed to run in parallel on a range of high-performance computing resources as well as on desktop multi-core machines. Cardiac cellular models are not sufficiently complex to make simulating a single cell in parallel particularly worthwhile, but many applications require multiple independent simulations, whether for comparing different models or performing parameter sweeps. Chaste contains features specifically designed for performing such independent simulations in parallel, and these are exploited in the code accompanying this paper as a demonstration. When combined with the abilities described above to load models from CellML dynamically and address the same biological entity consistently in any model through ontological annotation, this gives a powerful toolkit for analysing and comparing models. One can parallelise execution of an arbitrary experiment over different parameter sets or models.

#### 2.3.1. Steady state

It is useful to get models into a state where they produce the same action potential on subsequent stimuli or “paces.” In mathematical terms, this is the *limit cycle* of the periodic orbits they are taking through state variable space over time. Because the models are deterministic, if the state variables at time “*t*” are the same as the state variables at time “*t* + pacing period,” then this suffices as a definition of the limit cycle and a specification of the state variables together with the pacing period defines the steady orbit. We call these limit cycles the “steady state” in this discussion, in contrast to the usual steady state for an ODE system (when the state variables have ceased to change over time).

Using such a limit cycle steady state for initial conditions for simulations is advantageous for a number of reasons, discussed further in a blog post (http://mirams.wordpress.com/2014/07/22/initial_conditions). We have implemented a simple algorithm in Chaste (the “SteadyStateRunner” class) to take an action potential model with a certain regular stimulus and use CVODE for rapid pacing.

Of course, it takes an infinite amount of time to *reach* a steady state. So in practice, we define the steady state as being achieved when the change in state variables between subsequent paces has become sufficiently small (note that regular alternans can also be detected by examining the difference in state variables over two paces). At present, we use the *L*^2^-norm of the change in state variables being less than 10^−6^; future work will examine whether a metric based on the number and relative sizes of the state variables is more useful. Typically, this criterion is reached in a few hundred paces, when changing pacing rate or varying maximum conductance parameters, for most models. Action potential durations are established to within 0.01 ms, relative to pacing for 10,000 paces, for the range of models we have examined to date.

Certain models do not approach a steady state, or they tend to an un-physiological steady state, usually because conservation of ions is not satisfied (Livshitz and Rudy, 2009). A warning is provided to the user if the algorithm hits a (user-defined) maximum number of paces in its search for steady state to provide information that the model may be exhibiting such behavior. Otherwise, the model is returned to the user with its state variables in steady state for its stimulus.

## 3. Results

Here, we investigate the success rate of CellML conversion in Section 3.1, then discuss single cell simulation benchmarking in Section 3.2. We examine how features of tissue simulations can alter the choice of ODE solver in Section 3.3 and finish with a discussion of some numerical considerations in Section 3.4. All of the code required to reproduce the study and figures in this article has been made open source; it can be downloaded as a bolt-on project for Chaste v3.3 from https://chaste.cs.ox.ac.uk/q/paper/Frontiers2014, where an annotated tutorial-style walk-through of all the code can also be found.

### 3.1. Model conversion

The Chaste source repository includes a collection of CellML files obtained from the official CellML repository and annotated to work with Chaste. At present, it contains 75 model files that can successfully be converted with our tools, and the majority of these can be simulated using CVODE, although two are numerically fragile and four do not produce action potentials with the stimulus hard-coded in the model. Seven models we have tried do not convert; however these are all due to errors in the CellML encoding, such as invalid physical units, missing parameters, or over-constrained equations.

A number of studies have been enabled due to the consistent interface to electrophysiology models that we have presented in this article. In Mirams et al. (2011, 2014), a range of models were subjected to the same parameter changes and pacing protocols to model drug-induced block of multiple ion channels. Pathmanathan et al. (2011) examined how a wide range of models exhibited different conduction velocity convergence properties when using different numerical schemes for tissue simulations. By examining a wide range of models, it was established that the sodium channel formulation and upstroke velocity in a single cell simulation could inform the likely error incurred by the different schemes in a tissue context. Walmsley et al. (2013) used the auto-generated interface to explore a range of altered conductivities in the action potential model of O'Hara et al. (2011) to predict likely changes to electrophysiology under the mRNA expression changes observed in heart failure. Finally, Cooper et al. (2011b) used the consistent interface to models to compare and contrast different model behaviors in the same experimental situation.

### 3.2. Benchmarking cell simulations

In order to benchmark the various solvers for each model, we chose a reference problem of a regular stimulus action potential simulation. Where the model is not self-exciting, we use the stimulus parameters of magnitude, start time, period, and duration as defined in the CellML file. For self-exciting models, the length of the simulation was set to 1000 ms.

To generate a converged “reference solution” for each model, we used CVODE with analytic Jacobians (where available), with tolerances of 10^−7^ (relative) and 10^−9^ (absolute). The output timestep for the reference traces was set to 0.1 ms. Some of the methods we are using are implicit, and so relatively large timesteps could be taken whilst maintaining numerical stability. However, stability does not guarantee accuracy, and so we also need to ensure sufficiently small timesteps are taken in each method to get a reasonably accurate solution. To ensure a fair speed comparison, we first ran a test to establish timesteps (for the fixed-step methods) or tolerances (for CVODE) that would produce results within a common defined level of accuracy.

We chose a metric of Mixed Root Mean Square error (Marsh et al., 2012), a combination of relative and absolute errors, defined by:

(7) $$e_{\text{MRMS}}=\sqrt{\frac{1}{N}\sum_{t=1}^{N}{\left(\frac{{\hat{V}}_{t}\;-\;V_{t}}{1\;+\;|{\hat{V}}_{t}|}\right)}^{2}}.$$

**Figure 1:**
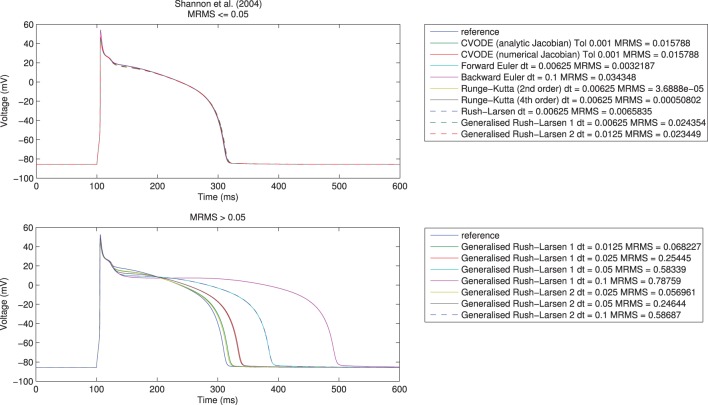
**Single cell action potential traces for the Shannon et al. (2004) model using a range of numerical algorithms with different timesteps**. **Top:** traces with *e*_MRMS_ ⩽ 0.05 (overlapping), **bottom:** traces with *e*_MRMS_ > 0.05. Note that nearly all the solvers in the upper figure diverged (failed to provide any solution) when running at larger timesteps or with more relaxed tolerances. It is the fact that the Generalized Rush–Larsen solvers are very stable that means that traces were produced even at large timesteps, so their dominance of the bottom plot should be seen as a demonstration of their stability rather than a statement on their accuracy.

Here, *t* indexes the voltage (*V*) samples at *N* distinct time points, $\hat{V}$ represents the reference solution, and *V* the test solution. We took *e*_MRMS_ ⩽ 0.05 as our acceptable error, as shown in Figure 1 for the Shannon et al. (2004) model. Across all model/solver combinations, this metric results in mean absolute errors of 0.3 ms in APD_90_, 0.8 ms in APD_50_, and 1.63 ms in APD_30_, 0.85 mV in peak voltage, 0.02 mV in resting potential, and 10 mV/ms in maximum upstroke velocity.

We first undertook the simulations with timesteps equal to 0.1 ms, ran the simulations, and calculated the resulting errors. If a simulation failed due to instability or the error criterion of *e*_MRMS_ ⩽ 0.05 was not met, then we halved the timestep (for fixed-step methods, or tightened the tolerances by ten-fold for CVODE) and repeated. Reassuringly, all of the solvers converged toward the reference solution for all of the models, although for some models not all solvers met the convergence criterion before the limit of 12 refinement steps was reached. We give an example of some traces generated using the different solvers in Figure 1. Notably, many of the explicit solvers were unstable at larger timesteps and immediately satisfied the error metric on the first timestep that produced a solution—clearly care must be taken to check the stability of these solvers for a given problem. On the other hand, solvers such as the Generalized Rush–Larsen methods are very stable but can give highly inaccurate answers when using large timesteps.

Hence, we recorded a suitable timestep/tolerance for each solver and model combination to meet the *e*_MRMS_ ⩽ 0.05 criterion and used these timesteps in a subsequent timing test. The timesteps that were used are presented in Tables 1, 2, ranked in terms of the computing time required from low to high. We infer that the models operating on the faster timescale require finer tolerances and timesteps, and that these are generally linked with longer simulation times (although the number of ODEs and pacing rate also play a role).

**Table 1:** **Timesteps and tolerances required to meet the error bound *e*_MRMS_ ⩽ 0.05 for all of the cell models in this study**.

**Rank**	**Model**	**CVODE**	**CVODE**	**F**	**B**.	**RK**	**RK**	**RL**	**GRL**	**GRL**
		**AJ**	**NJ**	**Euler**	**Euler**	**2nd**	**4th**		**1st**	**2nd**
		**(Tol.)**	**(Tol.)**	***n***	***n***	***n***	***n***	***n***	***n***	***n***
1	Noble (1962)	10^−3^	10^−3^	3	3	0	0	5	5	5
2	McAllister et al. (1975)	10^−3^	10^−3^	4	4	4	3	5	5	5
3	Luo and Rudy (1991)	10^−3^	10^−3^	4	0	4	3	0	0	0
4	Noble and Noble (1984)	10^−3^	10^−3^	0	1	0	0	2	3	3
5	Hilgemann and Noble (1987)	10^−3^	10^−3^	1	0	1	1	1	0	0
6	Earm and Noble (1990)	10^−3^	10^−3^	1	0	1	1	1	1	1
7	DiFrancesco and Noble (1985)	10^−4^	10^−4^	2	1	1	1	3	3	3
8	Stewart et al. (2009)	10^−3^	10^−3^	3	2	3	3	3	3	3
9	Beeler and Reuter (1977)	10^−3^	10^−3^	2	0	3	2	0	0	0
10	Paci et al. (2013) (atrial)	10^−3^	10^−3^	1	1	2	2	2	3	3
11	Noble et al. (1989)	10^−3^	10^−3^	2	2	0	0	2	3	3
12	Hodgkin and Huxley (1952)	10^−4^	10^−4^	1	3	1	1	1	2	2
13	Noble et al. (1991)	10^−3^	10^−3^	1	0	1	1	0	0	0
14	Dokos et al. (1996)	10^−3^	10^−3^	1	1	1	1	1	3	3
15	Sakmann et al. (2000)	10^−3^	10^−3^	1	0	1	1	0	0	0
16	Priebe and Beuckelmann (1998)	10^−3^	10^−3^	4	1	5	4	3	1	1
17	Paci et al. (2013) (ventricle)	10^−3^	10^−3^	3	0	4	3	3	1	—
18	Zhang et al. (2000)	10^−3^	10^−3^	0	1	0	0	1	2	2
19	Noble et al. (1998)	10^−3^	10^−3^	1	0	1	1	2	2	2
20	Noble and Noble (2001)	10^−3^	10^−3^	1	0	1	1	1	1	0
21	Maleckar et al. (2008)	10^−3^	10^−3^	1	0	1	1	1	1	1
22	Fox et al. (2002)	10^−3^	10^−3^	5	1	5	4	1	0	0
23	Courtemanche et al. (1998)	10^−3^	10^−3^	3	1	3	3	2	2	2
24	Pandit et al. (2001) (endo)	10^−3^	10^−3^	8	1	8	7	8	2	2
25	Maltsev and Lakatta (2009)	—	10^−3^	3	—	3	2	3	4	4
26	Kurata et al. (2002)	10^−3^	10^−3^	2	1	2	2	3	4	4
27	Iribe et al. (2006)	10^−3^	10^−3^	1	0	1	1	0	1	0
28	Nygren et al. (1998)	10^−5^	10^−5^	2	1	1	2	2	2	2
29	Espinosa (1998)	10^−4^	10^−4^	2	2	1	1	0	1	1
30	ten Tusscher et al. (2004) (endo)	10^−3^	10^−3^	6	0	6	6	0	0	0
31	Benson et al. (2008)	—	10^−3^	4	—	4	3	3	2	2
32	Fink et al. (2008)	10^−3^	10^−3^	6	0	6	6	2	2	1
33	ten Tusscher et al. (2004) (epi)	10^−3^	10^−3^	6	0	6	6	0	0	0
34	ten Tusscher et al. (2004) (M)	10^−3^	10^−3^	6	0	6	6	0	0	0

*Note that a printing/sampling time of 0.1 ms was used in the timestep selection test, so timesteps are presented by the number of times 0.1 was halved to create the ODE timestep; i.e., ODE timesteps for the fixed-step methods are of the form* 0.1/2*^n^ ms, so *n* = 0 represents 0.1 ms timestep, *n* = 1 represents 0.05 ms, *n* = 2 is 0.025 ms, etc. The CVODE relative tolerance that was required is shown (an absolute tolerance 100 times smaller was also set). In this table, the first half of the models are shown (fastest to simulate), the other half are in **Table 2***.

**Table 2:** **Timesteps and tolerances required to meet the error bound *e*_MRMS_ ⩽ 0.05 for all of the cell models in this study**.

**Rank**	**Model**	**CVODE**	**CVODE**	**F**.	**B**.	**RK**	**RK**	**RL**	**GRL**	**GRL**
		**AJ**	**NJ**	**Euler**	**Euler**	**2nd**	**4th**		**1st**	**2nd**
		**(Tol.)**	**(Tol.)**	***n***	***n***	***n***	***n***	***n***	***n***	***n***
35	Hund and Rudy (2004)	—	10^−3^	4	—	4	3	4	4	4
36	Demir et al. (1994)	10^−3^	10^−3^	3	3	1	1	4	6	4
37	ten Tusscher and Panfilov (2006) (M)	10^−3^	10^−3^	6	0	6	6	0	0	0
38	ten Tusscher and Panfilov (2006) (endo)	10^−3^	10^−3^	6	0	6	6	1	0	0
39	ten Tusscher and Panfilov (2006) (epi)	10^−3^	10^−3^	6	0	6	6	0	0	0
40	Grandi et al. (2010) (epi)	10^−3^	10^−3^	4	0	4	4	4	4	3
41	Grandi et al. (2010) (endo)	10^−3^	10^−3^	4	0	4	4	4	4	3
42	Mahajan et al. (2008)	10^−3^	10^−3^	4	1	4	4	2	5	3
43	Pandit et al. (2001) (epi)	10^−5^	10^−5^	4	3	9	8	4	3	3
44	Shannon et al. (2004)	10^−3^	10^−3^	4	0	4	4	4	4	3
45	Livshitz and Rudy (2007)	—	10^−4^	4	—	4	4	3	3	3
46	Davies et al. (2012)	—	10^−3^	5	—	5	4	3	3	3
47	Viswanathan and Rudy (1999)	10^−3^	10^−3^	4	7	4	4	3	0	0
48	Aslanidi et al. (2009b)	—	10^−3^	3	—	3	3	0	0	0
49	Matsuoka et al. (2003)	10^−3^	10^−3^	1	0	0	0	1	1	1
50	Carro et al. (2011) (endo)	10^−3^	10^−3^	4	0	4	4	4	5	3
51	Carro et al. (2011) (epi)	10^−3^	10^−3^	4	0	4	4	4	5	3
52	Faber and Rudy (2000)	10^−7^	10^−6^	8	9	7	6	8	9	8
53	Pásek et al. (2006)	10^−3^	10^−3^	7	4	7	7	7	—	10
54	Jafri et al. (1998)	10^−3^	10^−3^	8	6	8	8	8	6	3
55	Aslanidi et al. (2009a)	—	10^−3^	4	—	4	4	1	0	0
56	Winslow et al. (1999)	10^−5^	10^−5^	10	7	10	10	10	11	8
57	Wang and Sobie (2008)	10^−3^	10^−3^	3	0	3	3	3	1	1
58	Corrias et al. (2011)	10^−6^	10^−6^	8	8	4	6	8	8	8
59	Li et al. (2010)	10^−3^	10^−3^	9	0	9	8	9	4	3
60	Iyer et al. (2004)	10^−3^	10^−3^	11	6	11	10	11	11	8
61	Iyer et al. (2007)	10^−3^	10^−3^	10	1	10	9	10	9	7
62	O'Hara et al. (2011) (endo)	10^−3^	10^−3^	2	2	2	1	2	2	2
63	O'Hara et al. (2011) (epi)	10^−3^	10^−3^	2	2	2	1	2	2	2
64	Decker et al. (2009)	10^−3^	10^−3^	4	0	4	4	0	2	1
65	Pásek et al. (2008)	10^−3^	10^−3^	9	7	9	9	9	8	9
66	Bondarenko et al. (2004) (septal)	10^−3^	10^−3^	10	4	10	10	10	5	4
67	Bondarenko et al. (2004) (apical)	10^−3^	10^−3^	9	4	9	9	9	4	3
68	Clancy and Rudy (2002)	10^−3^	10^−3^	1	1	1	1	1	1	1

*Note that a printing/sampling time of 0.1 ms was used in the timestep selection test, so timesteps are presented by the number of times 0.1 was halved to create the ODE timestep; i.e., ODE timesteps for the fixed-step methods are of the form* 0.1/2*^n^ ms, so *n* = 0 represents 0.1 ms timestep, *n* = 1 represents 0.05 ms, *n* = 2 is 0.025 ms, etc. The CVODE relative tolerance that was required is shown (an absolute tolerance 100 times smaller was also set). In this table, the second half of the models are shown (slowest to simulate), the other half are in Table 1*.

The timing test consisted of approximately 5–10 s of run time (we adjusted simulation time according to how long a single pace took whilst calculating suitable timesteps). The reported time is therefore given in “wall time” (that is, the time it took for the computer to solve the ODEs) for a second of simulated cellular electrophysiology. The test was repeated three times in quick succession, and the time taken for the fastest of these three runs was recorded. The timing tests were run with different compilers, with and without lookup tables, on one machine (a 12 core Ubuntu 12.04.5 server with Intel® Xeon® X5650 2.67 GHz processors and 48 GB of RAM).

#### 3.2.1. Benchmarking compilers

We ran all of the timing tests under the following compilers: GCC (“debug”); GCC (optimized, “GccOpt”); GCC (optimized specifically for the current processor, “GccOptNative”); “Intel”; Intel production quality (“IntelProduction”); and Intel production quality with CVODE recompiled in the same way (“IntelProductionCvode”).

**Figure 2:**
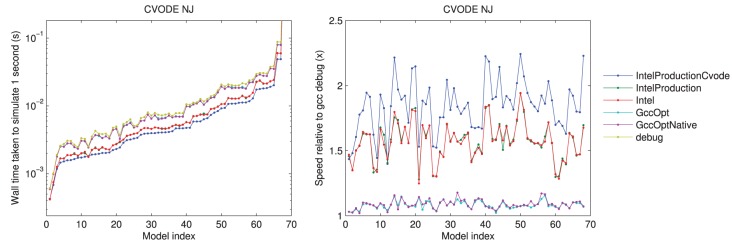
**Left:** a comparison of the time taken to simulate one second of activity with CVODE and numerical Jacobians, for each model, under different compilers. The Intel/IntelProduction mostly coincide, as do the GccOpt/GccOptNative lines. The last point (Clancy and Rudy, 2002) is omitted for clarity but shows the same trend at wall times around 4.65 s. **Right:** the speed-up provided relative to gcc (debug). Models ranked according to the wall time required under the IntelProductionCvode build, as listed in Tables 1, 2.

The results using different compilers are shown in Figure 2 for the CVODE numerical Jacobian solver. We observe little speed-up when using GccOpt or GccOptNative compilers. The main reason for this is that the pre-packaged CVODE library in Ubuntu that we used appears to have been compiled with GccOpt; for the non-CVODE solvers, the speed-up with GccOpt/GccOptNative was typically about 40%. The Intel compiler is certainly worth using if available, giving around 50% speed-up. For non-CVODE solvers, the speed-up using Intel was around 200–250%. We found a further performance boost could be gained for CVODE solvers by recompiling the CVODE library using the Intel compiler under production settings, taking us from around 1.5× to 2× as fast as non-Intel compilers. The rest of the results that are presented therefore use the Intel production build, with CVODE compiled using the same settings, unless otherwise stated.

#### 3.2.2. Numerical methods

**Figure 3:**
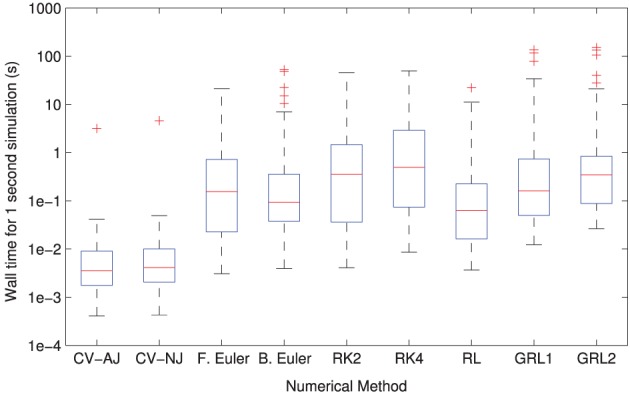
**Distributions of model simulation times for one second of electrophysiology for each different numerical method**. The CVODE solvers (“CV–AJ” with analytic Jacobian, and “CV–NJ” with numerical approximation to the Jacobian) consistently outperform any other method by an order of magnitude or more.

The distributions of solver times can be seen in Figure 3. CVODE consistently outperforms any of the fixed timestep methods, achieving the required accuracy typically 10–100× faster than the other solvers. This is consistent with our earlier findings (Spiteri and Dean, 2008, 2010). The only model where this is not the case is Clancy and Rudy (2002) where, for reasons we have not yet established, CVODE underperforms the fixed timestep methods.

**Figure 4:**
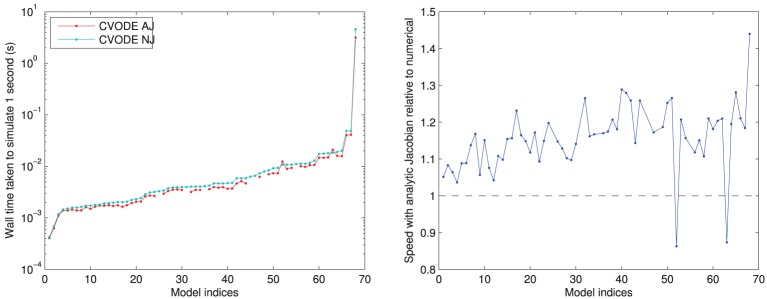
**Left:** performance of CVODE with and without use of an analytic Jacobian. For seven models an analytic Jacobian was not stable, and these points are omitted (those shown in Tables 1, 2 as “—” in the CVODE AJ column). **Right:** relative speed when using analytic Jacobian rather than a numerical approximation; 10–20% speed-up is most common. Models ranked according to the wall time required for CVODE NJ under the IntelProductionCvode build, as listed in Tables 1, 2.

In Figure 4, we compare the speed of the two versions of CVODE, AJ with analytic Jacobian provided and NJ where CVODE defaults to a numerical approximation. The use of analytic Jacobians provides between 10–20% speed-up for the vast majority of models. However, it is not usable for a few models, as mentioned above, and in two cases causes a slowdown; the numerical Jacobian is more consistent.

The two models where an analytic Jacobian causes a slowdown are Faber and Rudy (2000) and O'Hara et al. (2011) (but only its epicardial variant). These findings were replicated on all the different compilers, increasing our confidence that they are not spurious, and there is some feature of these models that means the analytic Jacobian does not speed up the solving. This could be related to the numerical issues that mean some analytic Jacobians lead to instabilities; we discuss this in Section 3.4.

#### 3.2.3. Lookup tables

**Figure 5:**
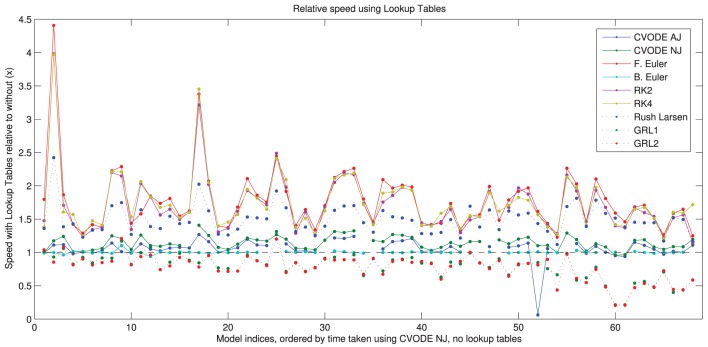
**Speed of simulation when using lookup tables, relative to without lookup tables**. Shown for all solvers with the “IntelProductionCvode” build. The black dashed line represents the same speed as without lookup tables; above is a speed-up; below is a slowdown. There are some gaps in the CVODE graphs to denote when analytic Jacobians are not available/stable (7 models), and also where the simulations with lookup tables failed to converge (2 additional models with analytic Jacobians, and 3 models with numerical Jacobians). Models ranked according to the wall time required for CVODE NJ under the IntelProductionCvode build, as listed in Tables 1, 2.

In Figure 5, we show the speed-up that is gained by using the different solvers with the lookup tables described in Section 2.2.4. More speed is gained with the numerical Jacobian (median 14%) than the analytic Jacobian (median 7%) despite lookup tables also being used for the analytical Jacobian calculation. It is likely that the extra calls to the right-hand side of the ODE system when using the numerical approximation, and possibly the numerical issues discussed in Section 3.4, outweigh this factor. There are ~60% speed-ups for the explicit methods and ~45% for Rush–Larsen. The backward Euler solver shows no difference because lookup tables are enabled by default when using this method. There is a slowdown to ~80% of usual speed for the Generalized Rush–Larsen solvers, caused by the way in which the generated C++ code is arranged. For clarity and to avoid scope collision, individual (partial) derivatives are computed within their own methods, i.e., the table lookup calculations are replicated once for each state variable in the model, rather than being shared as in other solvers. We intend to resolve this anomaly as part of the work to use symbolic expressions for these derivatives.

The use of lookup tables introduces further errors to the simulation (Cooper et al., 2010). For 13 model/solver combinations, using a lookup table just on *V* caused simulation failure because the voltage went outside the physiologically plausible range used for the table. In two further cases, using lookup tables caused the error to exceed our 5% threshold. The speed-up seen when using a table on cytosolic calcium too was very similar; in this case 14 combinations failed to simulate, but none exceeded the error threshold. Intriguingly, in many cases using lookup tables with CVODE *decreased* the error metric, sometimes considerably; in other cases it increased but remained below the threshold. Presumably the error is within CVODE's tolerances and the linear approximation introduced can have a corrective effect. For explicit solvers with the default settings, the error change was below 0.01% in almost all cases; where this was not the case the error was generally orders of magnitude below the threshold.

### 3.3. Benchmarking tissue simulations

To evaluate different ODE solver performance in a tissue simulation context, we chose a sample of seven models spanning the range of single-cell solving times that were reported in Section 3.2.

We used Chaste's default tissue simulation capabilities, which use the numerical methods described in Pathmanathan et al. (2010). The Chaste solvers were recently the subject of a thorough code verification study where the results of mono- and bi-domain simulations were shown to agree with analytic solutions (Pathmanathan and Gray, 2014).

For each action potential model, we set up a 1D monodomain problem, simulating activity for *t* ϵ [0, 500] ms on a 1D strand *x* ϵ [0, 1] cm, with conductivity of 1.75 mS/cm, and a stimulus of −30,000 μA/cm applied in *x* ϵ [0, 0.1] cm when *t* ϵ [1, 5] ms. This is sufficient for an action potential to traverse the whole domain. First, a convergence study was performed with varying mesh resolution and PDE timesteps (see the MATLAB code examine_reference_convergence.m in the Chaste project for all the results). This led us to choose mesh and time resolutions that gave reasonably well-converged answers and are of similar magnitudes to those commonly used in cardiac electrophysiology simulations. We selected two representative situations: a finite element mesh inter-node spacing of 0.01 cm (100 μm, 101 nodes) with PDE timestep of 0.01 ms; and the same mesh with a PDE timestep of 0.1 ms.

The reference traces for each case were produced using CVODE and analytic Jacobians, with relative and absolute tolerances of 10^−7^ and 10^−9^ respectively, without lookup tables, and with re-initialisation of CVODE on each PDE timestep. Again, we calculated the ODE timesteps/tolerances that were required to produce *e*_MRMS_ ⩽ 0.05 for the voltage trace at the final node (the last to be activated), with sampling timesteps of 0.1 ms.

**Figure 6:**
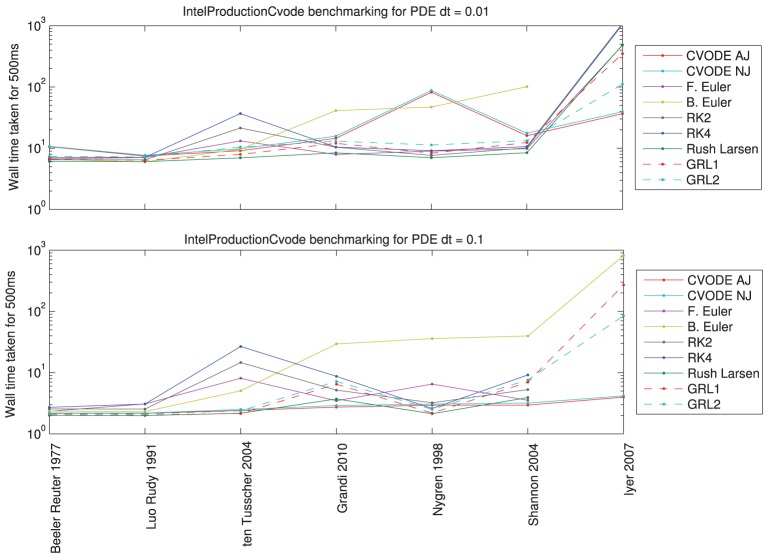
**Total wall time (including PDE solution, output, etc.) for a simulated 500 ms of monodomain activity in a 1 cm strand of tissue with inter-node spacing of 0.01 cm, using the fastest compiler settings**. **Top:** for a PDE timestep of 0.01 ms; **bottom:** for a PDE timestep of 0.1 ms. Missing data points indicate such small ODE timesteps were needed to reach a sufficiently converged solution that the simulation would have taken over 15 min (off the top of the scale).

Figure 6 shows the results of the solver benchmarking study. They are intricate, and we discuss the cases for a relatively fine PDE timestep (0.01 ms) and a moderately fine step (0.1 ms) separately below.

*For PDE timestep of 0.01 ms:* Simpler models are best solved using the Rush–Larsen, Generalized Rush–Larsen, and Backward Euler solvers. More complex models are still solved well with Rush–Larsen and even Forward Euler. Explicit methods (Forward Euler and second- and fourth-order Runge–Kutta) are never faster than Rush–Larsen. The Backward Euler method begins to become uncompetitive with models of the complexity of ten Tusscher et al. (2004) and higher. For the most complicated model (Iyer et al., 2007), CVODE performs much better than any other solver, and as expected the analytic variant provides a small additional performance boost.

*For PDE timestep of 0.1 ms:* Now the explicit solvers (Forward Euler, Runge–Kutta solvers) are not competitive for any model; there is always a better choice of solver. Simpler models are still solved well by Rush–Larsen and Generalized Rush–Larsen solvers. As before, the Backward Euler method does not perform well with models of ten Tusscher et al. (2004) and higher complexity, but in this case the difference is even more marked. Surprisingly, CVODE performs very well across the whole spectrum of models, with the larger PDE timestep giving the adaptive time-stepping the chance to “relax” when there is little activity and to therefore become very efficient. For a complicated model such as Iyer et al. (2007), CVODE is more than an order of magnitude faster than any other solver for the prescribed level of accuracy. For even larger PDE timesteps, we would expect CVODE to perform still better, whilst roughly the same amount of time would be taken solving the ODEs for the other numerical methods.

We confirmed that with our use of CVODE, described in Section 2.2.5, there is indeed no need in general to reset CVODE for each PDE timestep. The simulations with and without resetting display the same accuracy and convergence behavior. However, occasionally when resolving the upstroke for some cell models CVODE will fail to converge, especially when a very large PDE step is used. In this case, Chaste catches the error and performs a reset, and the simulation may then proceed.

Note that for all the models tested, the time taken to solve the ODEs dominates that taken to solve the PDE, consuming over 60% of the total run time for the simplest models, and over 99% for the most complex. In contrast solving the linear system arising from the PDE typically took less than 10% of the total time, and never more than 26%. The PDE solution becomes more challenging for 3D or bidomain problems. However, it is still the case that choosing a suitable ODE solving scheme is a problem worthy of attention. Although not presented here, we have used CVODE in large-scale tissue simulations with both the monodomain and bidomain equations and still observed a significant speed-up. The memory overhead, while present, has not been prohibitive when using a number of processors appropriate to obtain good parallel performance.

### 3.4. Numerical considerations

One requirement for adaptive solvers is that the maximum timestep that they can take is less than the duration of any square wave stimulus that is applied; otherwise stimuli can be missed completely if the RHS is not evaluated when the stimulus is “on.”

Cardiac cell models can be difficult to integrate, partly because some are composed of stiff systems of ODEs (Spiteri and Dean, 2010). Adaptive solvers such as CVODE can perform so well because they can refine the timestep as the different timescales of the system come into play.

Another problem is the presence of singularities: the values the right-hand side of the ODE system takes can be undefined (Inf or NaN), usually for certain specific values of *V*. Sometimes these are “removable singularities,” where the function tends to the same value from above and below. These singularities can be manually edited out by changing the CellML file, for instance using L'Hôpital's rule. Others have proposed solutions to this problem by using alternative expressions to capture gating kinetics (Hanslien et al., 2010).

Unfortunately, adaptive solvers such as CVODE can be prone to finding these singularities because they refine timesteps near steep gradients. In this case, it is worth changing the absolute and relative tolerances of the CVODE solver, the default being (10^−5^, 10^−7^). Unfortunately, it is not clear whether refining or relaxing the tolerances is the best strategy, and we have observed both approaches working for different models. The reasoning is that by relaxing the tolerances the solver may be able to “skip over” any singularity because the solution is not as refined around the singularity. But on the other hand, we have observed more stability problems with lookup tables enabled and also saw that a more accurate solution, provided by tightening tolerances (or avoiding lookup tables), can prevent these problems.

## 4. Discussion

Chaste has a wide range of features for performing single-cell and tissue cardiac electrophysiology simulations. CellML files defining different models can be automatically converted, on the fly, into C++ programs, with a consistent interface in terms of variable names and units. A wide range of ODE solvers can then be used to perform simulations with these models.

This article has shown the performance of the different ODE solvers, and required timesteps for an accurate solution, for a wide range of models. The required timestep (or solver tolerance for adaptive solvers) is very much model dependent, and should be tested in each case. For single-cell simulations the best performance is usually achieved using the CVODE solver, using analytic Jacobians that are automatically calculated, and code compiled with the Intel compiler using production settings. In most cases using Chaste's auto-generated “lookup tables” (which replace expensive functions) will provide a further speed-up with minimal impact on accuracy.

In the case of tissue simulations, the most suitable solver is less clear because the maximum ODE timestep is typically constrained by the PDE timestep, and voltage is treated as a constant across the PDE timestep. The most suitable solver can change depending upon the PDE timestep that is being used and the tolerable level of error for a particular simulation. In these cases, different solvers could easily be tested with the model of interest by utilizing the open source code written for this paper.

The metadata tags that we have proposed for cardiac electrophysiology models allow automatic conversion of tagged CellML models to C++, including any necessary units conversions, to create a standardized model interface. We presently use a small repository of CellML files with added tags (https://chaste.cs.ox.ac.uk/q/cellml), in order to develop working tools quickly without waiting for a community consensus process. We plan to convert these simple “tags” into a more structured ontology, which will be more widely useful, allowing interactions such as “get the sum of all the transmembrane potassium currents” without having to know *a priori* the precise quantities that are present in each model. To ensure long-term benefit, we plan to gather community support for such an ontology, and to add corresponding metadata to the main Physiome Repository (https://models.physiomeproject.org/electrophysiology).

Within all of the simulations and code discussed in this paper, the “experimental protocol” applied to a cell model is hard-coded in C++. Although this gives great flexibility, it also means that the essential features of the experiment, from a biophysical perspective, are somewhat obscured by the need to express them in C++ using the Chaste classes. We have therefore been developing the “standardized interfaces” ideas expounded in this paper into the concept of “functional curation” (Cooper et al., 2011b), and implementing it as an add-on component for Chaste. This uses a special purpose syntax for describing complex ‘virtual experimental protocols’ (Cooper et al., 2014), inspired by SED-ML (Köhn and Le Novère, 2008), much as CellML provides a dedicated language for describing the cellular models.

The functional curation tools use the annotation and units conversion functionalities described in this paper to allow any protocol to be applied to any compatible model, i.e., one possessing the biology probed by the protocol. We may therefore easily compare how different models react under the same experimental scenario, as well as characterize the behavior of individual models in multiple scenarios. In Cooper et al. (2014), we argue that these approaches are a prerequisite for future advances in experiment automation, parameter fitting, and open reproducible science.

## Author contributions

All the authors contributed to the software implementation described, and wrote and approved the paper.

## Funding

Jonathan Cooper and Gary R. Mirams gratefully acknowledge research support from: the “2020 Science” programme funded through the EPSRC Cross-Disciplinary Interface Programme (EP/I017909/1) and supported by Microsoft Research; and Gary R. Mirams acknowledges support from a Sir Henry Dale Fellowship jointly funded by the Wellcome Trust and the Royal Society (Grant Number 101222/Z/13/Z). Raymond J. Spiteri acknowledges research support from Canada's National Science and Engineering Research Council and the MITACS Network of Centers of Excellence.

### Conflict of interest statement

The authors declare that the research was conducted in the absence of any commercial or financial relationships that could be construed as a potential conflict of interest.

## Supplementary material

An open source Chaste project Frontiers2014 is available from http://www.cs.ox.ac.uk/chaste/download.html, to work with Chaste v3.3. The interested reader can download it, run the simulations, and analyse the results as presented in this paper. Documentation for this code is available at https://chaste.cs.ox.ac.uk/q/paper/Frontiers2014, which explains how to install it, what it does, and how it works.
